# Comprehensive Molecular Profiling for Relapsed/Refractory Pediatric Burkitt Lymphomas—Retrospective Analysis of Three Real-Life Clinical Cases—Addressing Issues on Randomization and Customization at the Bedside

**DOI:** 10.3389/fonc.2019.01531

**Published:** 2020-02-07

**Authors:** Kristyna Polaskova, Tomas Merta, Alexandra Martincekova, Danica Zapletalova, Michal Kyr, Pavel Mazanek, Zdenka Krenova, Peter Mudry, Marta Jezova, Jiri Tuma, Jarmila Skotakova, Ivana Cervinkova, Dalibor Valik, Lenka Zdrazilova-Dubska, Hana Noskova, Karol Pal, Ondrej Slaby, Pavel Fabian, Sarka Kozakova, Jakub Neradil, Renata Veselska, Veronika Kanderova, Ondrej Hrusak, Tomas Freiberger, Giannoula Lakka Klement, Jaroslav Sterba

**Affiliations:** ^1^Department of Pediatric Oncology, University Hospital Brno and Faculty of Medicine, Masaryk University, Brno, Czechia; ^2^International Clinical Research Center, St. Anne's University Hospital, Brno, Czechia; ^3^Department of Pathology, University Hospital Brno and Faculty of Medicine, Masaryk University, Brno, Czechia; ^4^Department of Pediatric Surgery, Orthopedics and Traumatology, University Hospital Brno and Faculty of Medicine, Masaryk University, Brno, Czechia; ^5^Department of Pediatric Radiology, University Hospital Brno and Faculty of Medicine, Masaryk University, Brno, Czechia; ^6^Department of Pharmacology, Faculty of Medicine, Masaryk University, Brno, Czechia; ^7^Regional Centre for Applied Molecular Oncology, Masaryk Memorial Cancer Institute, Brno, Czechia; ^8^Central European Institute of Technology, Masaryk University, Brno, Czechia; ^9^Department of Oncological Pathology, Masaryk Memorial Cancer Institute, Brno, Czechia; ^10^Laboratory of Tumor Biology, Department of Experimental Biology, Faculty of Science, Masaryk University, Brno, Czechia; ^11^Childhood Leukaemia Investigation Prague, Department of Paediatric Haematology and Oncology, 2nd Faculty of Medicine, Charles University, Prague, Czechia; ^12^Faculty of Medicine, Masaryk University, Brno, Czechia; ^13^Centre for Cardiovascular Surgery and Transplantation, Brno, Czechia; ^14^CSTS Health Care, Toronto, ON, Canada

**Keywords:** Burkitt lymphoma, targeted therapy, precision medicine, theranostics, pediatric oncology

## Abstract

In order to identify reasons for treatment failures when using targeted therapies, we have analyzed the comprehensive molecular profiles of three relapsed, poor-prognosis Burkitt lymphoma cases. All three cases had resembling clinical presentation and histology and all three patients relapsed, but their outcomes differed significantly. The samples of their tumor tissue were analyzed using whole-exome sequencing, gene expression profiling, phosphoproteomic assays, and single-cell phosphoflow cytometry. These results explain different treatment responses of the three histologically identical but molecularly different tumors. Our findings support a personalized approach for patient with high risk, refractory, and rare diseases and may contribute to personalized and customized treatment efforts for patients with limited treatment options like relapsed/refractory Burkitt lymphoma.

**Summary:**

The main aim of this study is to analyze three relapsed Burkitt lymphoma patients using a comprehensive molecular profiling, in order to explain their different outcomes and to propose a biomarker-based targeted treatment. In cases 1 and 3, the tumor tissue and the host were analyzed prospectively and appropriate target for the treatment was successfully implemented; however, in case 2, analyses become available only retrospectively and his empirically based rescue treatment did not hit the right target of his disease.

## Introduction

Burkitt lymphoma is a highly aggressive mature B-cell lymphoma commonly associated with translocation of *MYC* gene. The disease is classified as sporadic, endemic, or immunodeficiency related. In pediatric oncology, current standard intensive chemotherapy with anti-CD20 antibody regimens achieve long-term, disease-free survival in almost 95% of patients (1). However, a subset of patients who do not respond to the first-line chemotherapy and who experience relapse have very poor prognosis despite high-dose chemotherapy followed by stem cell transplantation (2). This subset of patients, for whom further chemotherapy-based therapies are futile, is recently often considered for therapies based on molecular analysis of their tumor tissue. We present three cases of relapsed Burkitt lymphoma. Cases 1 and 3 were treated with a therapy that reflected the molecular signature of the child's tumor, but in case 2, the therapy “missed” the target because his molecular signature was not known at the time retrieval therapy was initiated. The findings suggest that molecular signatures are unique, and a tissue biomarker-based customized therapy may be the better approach to address these poor prognosis patients than just another biomarker agnostic randomized trial.

## Methods

A comprehensive molecular profiling consisted of whole-exome, gene expression profiling and a profile of phosphorylated proteins and single-cell phosphoflow cytometry of three cases of relapsed pediatric Burkitt lymphoma searching for biological rationale for different responses to the therapy and different clinical outcomes.

### Whole-Exome Sequencing

Whole-exome sequencing (WES) using the TruSeq DNA Exome Kit, the NextSeq 500/550 Mid Output Kit v2.5, and a NextSeq 500 sequencing device (all Illumina, CA, USA) was done in all three cases. Input material was 400 ng of DNA obtained from the peripheral blood (for germline exome) and formalin-fixed, paraffin-embedded (FFPE) tumor sample with ≥20% cancer cell count measured in the surface area of tissue slides for somatic exome. WES was done with high coverage where at least 90% of targeted regions were covered 20 times.

### Gene Expression Profiling (Transcriptome Examination)

Gene expression profiling using the Affymetrix GeneChip Human Gene 1.0. ST Array (Applied Biosystems, CA, USA) was done in all three cases. Input material was 250 ng of RNA obtained from frozen tumor tissue. Samples were prepared using the GeneChip WT PLUS Reagent Kit (Affymetrix, CA, USA) according to the manufacturer's protocol. Subsequently, chips were hybridized using the GeneChip Hybridization Oven, washed using the GeneChip Fluidics Station, and scanned on the GeneChip Scanner (all Affymetrix, CA, USA), and CEL files were generated. Data were processed using R software version 3.3.3 (3). Gene expressions of 220 selected genes were subsequently compared to accumulated normal tissue samples as described previously (4), utilizing two comparator sets: one consisting of 408 normal tissue samples of different diagnoses (main general comparator) and one consisting of 5 samples of normal germinal center B cells (complementary-specific comparator). Samples were downloaded from Gene Expression Omnibus and ArrayExpress databases, and names of the database samples are listed in Supplementary Material 1. Expression data were calculated as Robust Multichip Average (RMA) with background correction and quantile normalization implemented in rma function in oligo package (5). Difference of expression of each gene was calculated as fold change (FC) from the mean of the comparator set and tested using a two-sided one-sample *t*-test, with false discovery rate (FDR) adjustment applied. An FC value of 0.5 and more was considered important. No specific *p*-value was considered limiting the discrimination of differently expressed genes with FC > 0.5. Utilizing the general comparator consisting of 408 samples offers highly significant results corresponding to the power of 10 to −25 for the FDR-adjusted *p*-values for most of the evaluated genes with FC of 0.5 or more, and rising to the power of 10 to −100 for the FDR-adjusted *p*-values for genes with FC > 2.

RNA transcription data from the tumor tissues were analyzed as well using Biogrid (http://thebiogrid.org), and http://www.genome.jp/kegg/pathway.html and mathematical simulations of protein–protein interactions as described before (6).

### Profile of Phosphorylated Proteins

Human Phospho-RTK Array Kit (R&D Systems) was used to determine the relative levels of tyrosine phosphorylation of 49 different RTKs. Human Phospho-MAPK Array Kit (R&D Systems) was employed for the detection of phosphorylation status of 26 MAPKs, serine/threonine kinases, and other signaling proteins. Both arrays were performed as previously described (7).

### Single-Cell Phosphoflow Cytometry

Peripheral blood mononuclear cells (PBMCs) were separated on Ficoll-Paque (GE Healthcare) according to the manufacturer's instructions. PBMCs were reconstituted in a culture medium consisting of RPMI 1640 with 25 mM HEPES, L-glutamine, 100 U/ml penicillin, and 100 mg/ml streptomycin (Lonza, Basel, Switzerland) to a final concentration of 2 million cells per milliliter. After a 1-h rest at 37°C in a 5% CO_2_ atmosphere, the cells were stimulated on 96-well plate containing coated anti-CD3 (10 μg/ml, Exbio Praha) and free costimulatory anti-CD28/CD49d antibodies (1 μg/ml, BD Biosciences) for 5, 15, and 30 min. The cells were fixed with 4% formaldehyde for 10 min and permeabilized with ice-cold methanol for 30 min. The following fluorochrome conjugates were used for cytometric detection: phospho-Akt (Ser473)-Alexa Fluor 488, phospho-S6 (Ser235/236)-Pacific Blue (Cell Signaling Technologies), phospho-mTOR (Ser2448)-PE (eBioscience, Thermo Fisher), CD45-Pacific Orange, CD45RA-APC (Exbio), CD8-PE-Cy7 (Beckman Coulter), CD4-PerCP-Cy5.5, and CD3-APC-H7 (BD Biosciences). The samples were acquired on Canto II flow cytometer and analyzed using FlowJo software (BD Biosciences).

## Results

### Case 1

A 7-year-old previously healthy boy presented with *t*_(8;14)_ positive abdominal stage III Burkitt lymphoma (St. Jude staging system). The boy was initially treated as per the standard BFM B-NHL Registry 2012 protocol with the addition of rituximab according to the most recent published literature (1). He responded well to the therapy and achieved a very good partial response after two cycles. His clinical course was complicated by an episode of duodenal obstruction/intussusception requiring surgical intervention. The histology from this resection revealed sclerosing mesenteritis with no evidence of lymphoma, congruent with the conclusion of a study using ^18^F-fluorodeoxyglucose positron emission tomography/computed tomography (FDG PET/CT) that revealed a very small residual tumor with only borderline FDG PET avidity. Unfortunately, the patient had disease progression 6 weeks following the completion of protocol therapy (and 3 months from the second surgery) with a new lesion within the tumor resection margin and a new mediastinal mass. A biopsy of the abdominal lesion confirmed the recurrence of Burkitt lymphoma with persistent areas of sclerosing mesenteritis.

As sclerosing mesenteritis has been associated in the literature not only with B-cell lymphomas but also with activation of the PI3K-delta pathway and immunodeficiency (8, 9), a candidate testing for this specific mutation was performed.

**Figure 1:**
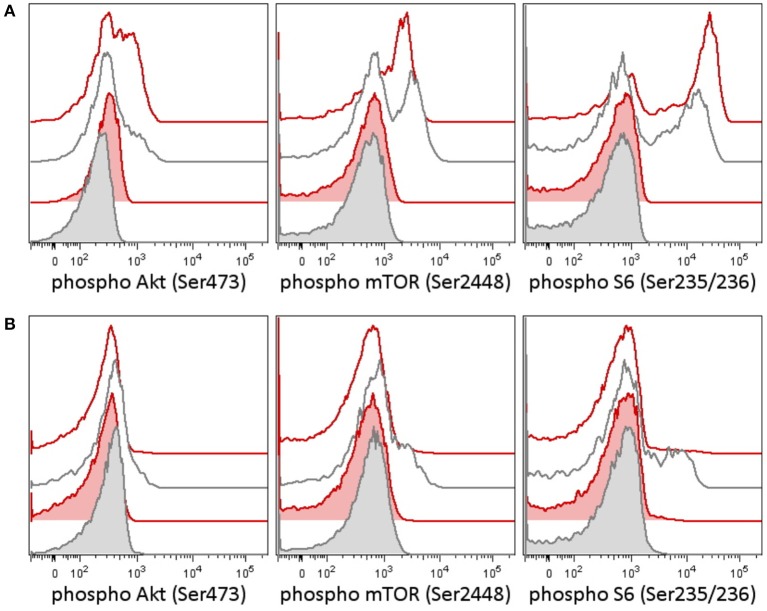
Phosphorylation patterns in the PI3K pathway in peripheral blood T-lymphocytes before **(A)** and after **(B)** therapy in case 1. Case 2 patient had a germline variant of PIK3CD, which was present in the tumor as well. Peripheral blood T-lymphocytes (patient 1's lymphocytes contained only T cells at the time of testing) were tested for activation of the PI3K signaling pathway [reflected as a phosphorylation of Akt (Ser473), mTOR (Ser2448), and S6 ribosomal protein (Ser235/236)] before and following therapy. **(A)** Patient T-lymphocytes showed increased basal phosphorylation of Akt as well as increased phosphorylation of Akt and S6 upon T-cell receptor (TCR) stimulation before treatment compared to an independent healthy control (the result is representative of three independent tests). **(B)** A week following the addition of idelalisib (a PI3K inhibitor), to the patient's therapy, the phosphorylation of Akt, mTOR, and S6 dropped down. CD3+ T-lymphocytes are shown in basal state (tinted histograms) and 15 min upon anti-CD3/CD28/CD49d stimulation (blank histograms). Red, patient 1; black, healthy control.

In the tumor, there was proven disruption of MYCC and IgH in 97% of cells according to fluorescence *in situ* hybridization (FISH). Karyotype of the tumor showed 46 chromosomes with complex changes. A germline variant of c.935C>G (p.S312C) in the PI3K-delta subunit was found both in the child and in the father. The patient's older sister and mother were negative for this variant. We tested the intracellular signaling downstream of PI3K using flow cytometry assessment of phosphorylation of Akt, mTOR, and S6 proteins in the patient's peripheral blood T-lymphocytes and detected increased basal and T-cell receptor (TCR)-induced activation (Figure 1A). Similarly, increased levels of PI3K were confirmed by RNA transcriptome analysis of the tumor tissue with Affymetrix GeneChipST 1.0. This analysis also revealed an increased expression of HR23B, a predictor of response to histone deacetylase (HDAC) inhibitors. Immunohistochemistry revealed a strong expression of PD-1L. The variant p.S312C has been described previously as mutation in brain cancer cell line and prostate cancer cell line (10) but has been classified as benign for development of immunodeficiency according to the ClinVar database. The allele frequency ranges between 0.008 and 0.030 in population databases (gnomAD 0.02, ExAc 0.0217, 1000G/ALL 0.008, 1000G/EUR 0.029) and was found to be 0.018 in our cohort of 508 cord blood samples (not published). Thus, this variant cannot be considered pathogenic. However, it may predispose the PI3K pathway to be activated, if other genetic and/or non-genetic factors are present.

Interestingly, even though the biopsy at the time of initial diagnosis had been tested for *TP53* and no alteration of the gene was found, in the biopsy obtained from the relapse, a new *TP53* R273C somatic mutation was identified in the tumor.

Retrieval therapy was administered with obinutuzumab 550 mg/m^2^, ibrutinib 140 mg/m^2^, and two cycles of ifosfamide, carboplatin, and etoposide (ICE) chemotherapy. The patient had further progression on this therapy, and a more molecular biomarker-driven theranostic approach was discussed. The therapy was changed to a single-agent window using a specific inhibitor of PI3K idelalisib 200 mg/m^2^/d. In 2 weeks, we were able to document a markedly decreased PI3K pathway activation in the patient's peripheral blood T-lymphocytes (Figure 1B), but the disease was still showing further radiological progression. Therapy with idelalisib was not discontinued, and ibrutinib 140 mg/m^2^ daily was reintroduced. Based on the transcriptome analysis, valproic acid for HDAC inhibition aiming for serum levels of 80–100 μg/ml was added, and nivolumab at 3 mg/kg every second week and metronomic cyclophosphamide at 25 mg/m^2^/7 days on/7 days off were introduced for immune modulation. To support local disease management and support the tumor antigen presentation, the patient received 21-Gy radiation to the site of the abdominal relapse. There was evidence of partial remission on FDG PET/CT 3 months later and stable disease 6 months later. Due to persistence of a viable tumor on FDG PET/CT and high toxicity of allogenic stem cell transplant reported in nivolumab-treated patients (11), this approach was not considered as treatment of choice. Consequently, personalized immunotherapy with dendritic cell-based vaccine was preferred to support the antitumor immunity, and treatment with dendritic cells loaded with whole tumor lysate according to phase I/II protocol (EudraCT No. 2014-003388-39) (12) was initiated. The residual tumor resected after 11 months of such therapy consisted of mainly necrotic tissue with lymphocytic infiltration with no evidence of viable tumor. Considering that the child had achieved complete remission, valproic acid, ibrutinib, and idelalisib were gradually discontinued and the patient is continuing to take biweekly intradermal applications of autologous dendritic cell vaccine and nivolumab until May 2018 when all his 37 manufactured doses of dendritic cell-based vaccine were used up.

The progression-free survival (PFS) of 46 months following a customized, tumor tissue molecular analysis-guided regimen was the longest PFS this child had achieved. The comparison of his earlier therapies reveals that he had achieved PFS1 6 months on the initial standard BFM protocol, and PFS2 only 1 month on the intensive retrieval therapy using anti-CD20 (obinutuzumab), ICE, and ibrutinib. His individualized therapy was outpatient based, associated with minimal treatment-related toxicities and allowed the child to return to school and perform all activities of daily living.

### Case 2

A 3-year-old boy diagnosed abroad with widely disseminated Burkitt lymphoma (abdomen, bone marrow, and both kidneys) was initially treated with the same standard BFM-based chemotherapy, but without rituximab. Before the completion of the fifth cycle, the patient had disease progression with a biopsy-positive new lesion in the right cheek. He continued with a relapse ALL protocol/ALL-REZ BFM 2002 in his home country outside the Czech Republic. As no therapeutic response was achieved, he was referred to our institution for a second opinion and management. He received two cycles of R-ICE (rituximab, ifosfamide, carboplatin, etoposide) given as per the ANHL0121 protocol achieving partial response, but the treatment was accompanied with severe life-threatening toxicities. He underwent surgery to obtain specimen for theranostic testing; however, the amount of the tumor tissue was not sufficient for all molecular studies. Based on our previous success in case 1 and as bridging to high-dose chemotherapy, he therefore continued with ibrutinib 140 mg/m^2^ daily, idelalisib 100 mg/m^2^ daily, and cyclophosphamide 1.5 mg/kg daily week on/week off for 6 weeks. Due to toxicities of intensive therapies and a clinical need for further therapy as bridging to stem cell transplant, the targeted agents were in this case based on our previous experience and a literature review. Despite a high-dose carmustine, etoposide, cytarabine, melphalan (BEAM) chemotherapy as per the AHOD0121 protocol (13) and autologous stem cell transplant being performed, he continued to do poorly. The patient had disease progression 3 weeks after BEAM conditioning and autologous stem cell transplant with a new lesion in the abdomen and continued to progress with massive L3 blast presence in the cerebrospinal fluid. He died due to disease progression 11 months from the initial diagnosis and 6 months after his first progression.

### Case 3

A 12-year-old boy was diagnosed with bulky abdominal Burkitt lymphoma. The patient was initially treated as per the standard BFM B-NHL Registry 2012 protocol with the addition of rituximab, but he achieved only partial response after two cycles, and assessment after four cycles revealed residual tumor with still increased FDG PET avidity. Three months later, the FDG PET/CT showed radiological progression of the primary tumor and dissemination in the right retromandibular area and anterior mediastinum. The relapse of Burkitt lymphoma was confirmed by biopsy. However, WES from the relapsed tumor sample revealed high tumor mutation burden−31 mutations/Mb; moreover, gene expression profiling detected strong expression of PD1, and the overall expression patterns of the case 3 were very similar to case 2 patient with very high fibronectin expression. First, participation in the randomized ibrutinib retrieval trial was planned here; however, based on molecular profiling and our previous experience from case 2, we have prioritized immune therapy here. He achieved radiological partial remission after third R-ICE cycle and then continued with nivolumab single agent only. After 12 weeks of nivolumab, he achieved first complete remission. His first PFS on standard intensive protocol was 7 months, but the second PFS with using immunotherapy is 14 months.

### Analyses

Somatic exome analysis of relapse samples revealed variants in the *TP53* gene in cases 1 and 2 (p.R273C in case 1 and p.R248L in case 2, NM_000546). p.R273C and p.R248L in *TP53* have been previously described as loss of function mutations based on *in vitro* functional analyses (14–19). Somatic exome analysis in case 1 detected a number of variants; the selected ones are shown in Supplementary Material 2. Germline exome analysis in case 1 also confirmed p.S312C (NM_005026) variant in the *PIK3CD* gene in the heterozygous form. Somatic exomes of cases 2 and 3 revealed a number of variants; the selected ones are also available in Supplementary Material 2.

Gene expression profiles of all three cases proved to be very similar; the highest expressions showed genes involved in immune system (*BTK, CD79A, CD79B*, and *KLHL6*). In cases 1 and 2, increased expression also showed genes involved in DNA damage response (*BRCA1, BRCA2, FANCA*, and *FANCD2*). In case 1, *CSF1R* and *PDGFRA* genes were also found to be increasingly expressed, while no genes coding tyrosine kinases showed to be overexpressed in case 2. In case 3, increased expressions showed genes involved in fibroblast growth factor signaling. In comparison to other pediatric oncology patients analyzed at our institute, transcriptome analysis in cases 1 and 2 revealed significantly increased expression of the *MYC* proto-oncogene.

**Figure 2:**
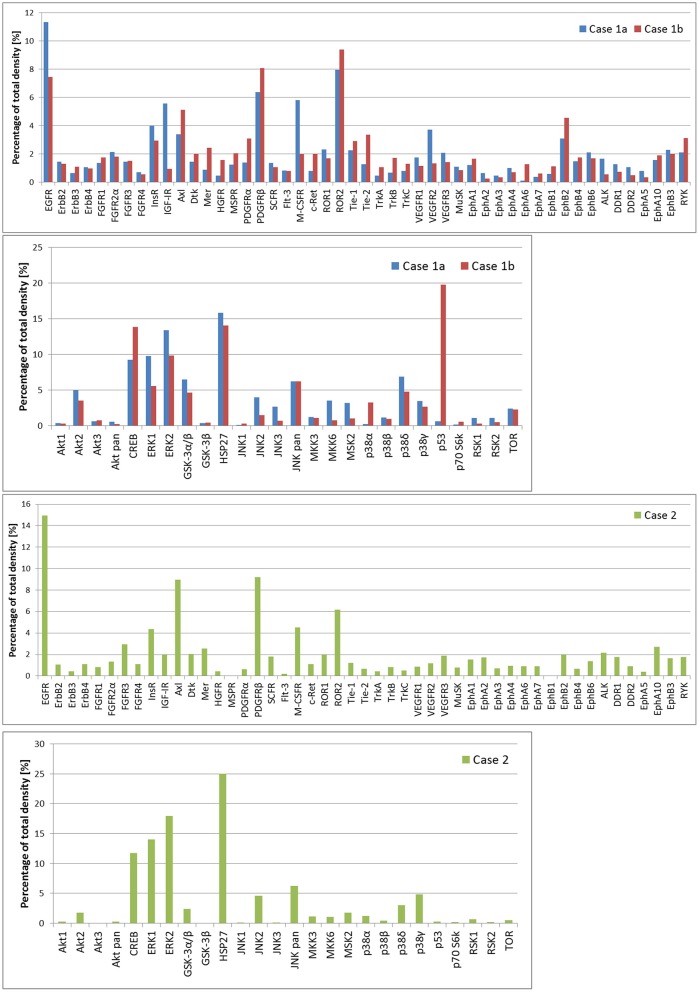
The relative phosphorylation analysis of tumor tissue samples. Human Phospho-MAPK Array Kit (R&D Systems) was employed for the detection of phosphorylation status of 49 RTKs, 26 MAPKs, serin/threonin kinases, and other signaling proteins, which performed using phosphoprotein arrays.

In case 1, two samples of the tumor tissue were also analyzed for activity of cell signaling pathways using phosphoprotein arrays for detection of RTKs, MAPKs, serine/threonine kinases, and other signaling protein as specified above: tumor tissue sample after the first line of treatment (Figure 2: case 1a) and second sample taken during the treatment of relapsed disease (Figure 2: case 1b). Phosphorylation profiles showed high relative activities of EGFR, PDGFRβ, ROR2, CREB, ERK1/2, and HSP27 in both samples. Furthermore, a very high level of phosphorylation was detected for p53 protein on Ser46 in the second sample in comparison to the first sample from this patient. This finding is in full accordance with the previous proapoptotic treatment including etoposide administration (20). In case 2, nevertheless, phospho-RTK analysis (Figure 2: case 2) revealed high phosphorylation of EGFR and PDGFRβ, and the phosphorylation profile of MAPKs, serine/threonine kinases, and other signaling proteins showed high activities of CREB, ERK1/2, and HSP27 in ascending order of density value.

Serology of Epstein–Barr virus (EBV) revealed the IgG positivity of EBV nuclear antigen (EBNA)-1 and the IgG positivity of viral capsid antigen (VCA) as well case 1 and case 2.

## Discussion

The introduction of highly intensive multiagent chemotherapy has dramatically improved the survival rates of primary childhood Burkitt lymphoma. While the initial treatment can have an over 90% success rate using standard intensive chemotherapy with rituximab, the outcome of children with relapsed Burkitt lymphoma is still very poor. The difficulties with treating chemotherapy-resistant relapsed tumors suggest an evolution of a more complex and more resistant disease (21), as could be documented by a new TP53 mutation in our case 1 at relapse, which was suggested by phosphoproteomic assay as well. The overview of our three cases reveals children with some very similar characteristics of their diseases, with alike pattern of cell signaling in tumor tissue, treated with identical agents in the first part of their relapse treatment, who experienced very dissimilar outcomes after the first relapse. It suggests that the tumors with similar histological features may harbor chemotherapy-resistant, genetically and biologically distinct subclones that become more dominant after intensive chemotherapy (21). At presentation, a fraction of these chemotherapy-resistant subpopulations may be small but, following intensive maximum tolerated dose-based chemotherapy, probably increases, and the tumor residuum is subsequently populated by resistant subclones. This evolution was furthermore evident on the evolution of molecular findings in the first patient and supports the need for a careful theranostic analysis and repeated biopsies whenever clinically indicated. Treatment of relapsed disease should be based on a detailed molecular analysis of the most recent available sample, i.e., at the time of relapse or progression rather than on original tumor biopsy only. The choice of drug combinations reflecting a broader molecular profile was based on reports that customized combinatorial therapies may produce more sustained responses (22, 23). Furthermore, as many biological agents are in fact chemotherapy sensitizers, their proper dosage should carefully be titrated to avoid severe systemic toxicity. In case 1, we have started with a single-agent idelalisib to target what was thought to be the driver mutation and gradually added additional targeted agents but at doses about 50% of those recommended in the Summary of Product Characteristics to avoid severe toxicity.

To successfully apply precision oncology principles into clinical practice, a requisite testing for molecular targets for each patient needs to be completed. As pointed above, while all three patients had histologically identical disease and were given the same combination of agents in the first- and two of them as second-line treatments, in case 2, we did not have a representative tumor sample timely available and his therapy was based only on detailed literature review and not the theranostic concept (24–26). The biology of the relapsed disease of case 3 reflected by transcriptome was similar to that of case 2, so a different approach could be undertaken, and while reflecting high mutational burden and increased expression of the PD-1L detected by immunohistochemistry and transcriptome, anti-PD-1 antibody was successfully used here.

While analyzing the transcriptomic results including considerations of gene and network interactions using https://string-db.org/ and http://www.genome.jp/kegg/pathway.html databases (6, 21), we were able to distinguish different patterns of tumor biology among our patients. Case 1 suggested neurotrophic receptor tyrosine kinase 1 (NTRK1) as a signaling protein and one of the best targets. In case 2 and case 3, in contrast, despite being clinically and histologically similar, transcriptomic results suggest an entirely different network, where fibronectin 1 (FN1) has a very complex downstream impact. Because FN1 is not a signaling protein and a druggable target, it is likely that we missed the putatively most important pathway in case 2. One may speculate that integrin inhibitors like cilengitide could be a better therapeutic option here. For case 3, FN1 seemed to be the key molecular hub as well, and it was one of the reasons for clinical decision to rely on tumor mutational burden and PD-1 ligand expression and treat the patient with immune therapies, rather than small molecules.

The localization of *MYC* proto-oncogene on q24 of the human chromosome 8 and its translocation to chromosome 14 is considered pathogenic in most cases of Burkitt lymphoma. In our cases, the RNA transcription analyses as described above indicate the activations of different sets of genes. These patients were almost identical in their clinical presentation, histology*, MYC* status, and initial clinical response to standard chemotherapy. Early clinical testing initiatives are beginning to employ individual profiles/fingerprint analyses to compile patients into histologically or biologically similar series (27), and as these efforts continue, new clinical trial designs will emerge (28, 29).

The research that has emerged over the last 40 years disproves the concept that cancer is a consequence of a single oncogenic change. It is widely accepted that an initiating oncogenic change such as translocation involving MYC is interpreted within the patient's genome, and further genomic alterations lead to the oncogenic inducers hijacking host-specific physiological responses such as angiogenesis, inflammation, and immune evasion. These normal physiological responses are not detected by DNA mutational analysis because they represent reactivation of developmentally silent pathways. We advocate the use of combinations of biological agents addressing not only the DNA mutations but also the normal physiological responses of the host as they are reflected in the individual's molecular signature reflected on transcriptomic and proteomic levels. In case 3, we successfully used immunotherapy reflecting the molecular profile of the tumor. In cases 1 and 2, we used a combination of ibrutinib (inhibitor of BCR signaling), idelalisib (direct PI3Kdelta inhibitor), valproate (HDAC inhibitor with potential to enhance responsiveness to immune therapies), and nivolumab (a host immune response modulator). Both patients were intended to receive an immune-supportive therapy using autologous dendritic cell vaccination with non-immune-suppressive maintenance agents such as checkpoint inhibitors, but only case 1 patient had achieved sufficient duration of the clinical response to live long enough to enable the preparation of his vaccine. Unfortunately, because we did not have the benefit of molecular information on genome or transcriptome in case 2, the therapy could not be customized enough to provide a more effective therapeutic combination. Our results revealing highly phosphorylated EGFR, PDGFRβ, ROR2, ERK1/2, or Hsp27 in all samples are also in accordance with previously published findings on Burkitt lymphoma (30, 31). Interestingly, activation of EGFR and ERK signaling via EBV oncoprotein LMP1 was also reported (32, 33) and our results thus concur with the latent EBV infection as suggested by serological analysis.

One of the most interesting observations was the discordance between laboratory and clinical responses to biomarker-based targeted therapy in case 1. Even though there was evidence of normalization of PI3K pathway activity, the evidence of radiological response was significantly delayed and gave an impression that the patient continued to progress. As has been frequently observed with biological therapies, the biomarker response may be more informative and preceded in this case the radiological response. While using biological therapies, we must allow sufficient time to pass before the patient is evaluated using present radiomorphological methods.

As we show, in cases where individualization of treatment protocols can be based on the recent molecular information, the likelihood of successful therapy may be increased, but the use of a targeted agent without laboratory evidence of contemporary target activation may not only lack benefit—it may even be harmful. Similarly, while treating sepsis, we are not using several-month-old microbiology results to guide antimicrobial treatment. Considering that there are presently numerous initiatives intending to study the addition of idelalisib and/or ibrutinib to existing retrieval therapies for relapsed and refractory mature B-cell lymphomas, it may be of value to collect enough samples for tumor tissue analysis and enable similar retrospective comparisons of patients who either failed or responded to therapy. An attractive concept inspired by our cases may be the successful sequence of different treatment modalities, such as intensive chemotherapy to debulk the initial tumor volume, followed by targeted biomarker-based treatment and stimulation of autologous immune response later on to consolidate the response.

## Conclusion

Precision medicine has significantly altered the practice of clinical oncology, but no standardized approach to the choice of these therapies exists. The three cases presented here emphasize that despite similarities in the presentation, histology, age, tumor site, and initial treatment response, the biology of tumors may differ significantly between cases and may change over time. Case 2 patient had an entirely different molecular signature and thus biology, without underlying relevant germline mutation, but such differences in molecular profile could be appreciated in retrospect only. We conclude that considering the dire outcomes of relapsed Burkitt lymphoma, theranostic testing may identify the most frequent molecular profiles that lead to therapeutic resistance and may help to improve frontline therapies sufficiently to prevent relapses and 1 day to replace our decade-old and toxic drugs like anthracyclines and alkylating agents.

## Data Availability Statement

The datasets for this article are not publicly available because it is not in accordance with our institutional policy. We handle data of rare entities that may be at risk of identification. Requests to access the datasets should be directed to Kristyna Polaskova, polaskova.kristyna@fnbrno.cz.

## Ethics Statement

The studies involving human participants were reviewed and approved by Ethics Committee for Multicenter Clinical Trials of the University Hospital Brno. Written informed consent to participate in this study was provided by the participants' legal guardian/next of kin.

## Author Contributions

KP wrote the draft of the manuscript and evaluated patient record. TM wrote the manuscript. DZ and AM evaluated patient records. MK did the statistical analyses. PMa, ZK, and PMu participated on the treatment decision and evaluated patient records. MJ performed pathological investigation. JT performed surgical procedures. JS and IC performed the radiological evaluations. DV, LZ-D, and SK participated on the manuscript. HN, KP, OS, PF, JN, RV, VK, OH, and TF performed biological samples analyses. GK supervised and wrote the manuscript. JS conceived and supervised the project and wrote the manuscript.

### Conflict of Interest

The authors declare that the research was conducted in the absence of any commercial or financial relationships that could be construed as a potential conflict of interest.
