# Supplementary material for: Comprehensive Molecular Profiling for Relapsed/Refractory Pediatric Burkitt Lymphomas—Retrospective Analysis of Three Real-Life Clinical Cases—Addressing Issues on Randomization and Customization at the Bedside
Source: Front Oncol. 2020 Feb 7;9:1531. doi: 10.3389/fonc.2019.01531 (PMC7027364; doi:10.3389/fonc.2019.01531)
Supplement: Supplementary file 1 [file Data_Sheet_1.pdf]

Supplementary Material 1

Control sample of 5 specific tissues

GSM1143706 germinal center B cells 1

GSM1143707 germinal center B cells 2

GSM1143708 germinal center B cells 3

GSM1143709 germinal center B cells 4

GSM1143710 germinal center B cells 5

Control backgroud of 408 normal tissues samples

GSM1427078_Muscle_E_1A.CEL

GSM1427081_Muscle_S_2A.CEL

GSM1427084_Muscle_C_3A.CEL

GSM1427087_Muscle_S_4A.CEL

GSM1427090_Muscle_E_6A.CEL

GSM1427093_Muscle_C_7A.CEL

GSM1427096_Muscle_S_8A.CEL

GSM1427099_Muscle_C_9A.CEL

GSM1427102_Muscle_S_10A.CEL

GSM1427105_Muscle_S_11A.CEL

GSM1427108_Muscle_C_12A.CEL

GSM1427111_Muscle_S_13A.CEL

GSM1427114_Muscle_E_14A.CEL

GSM1427117_Muscle_C_15A.CEL

GSM1427120_Muscle_E_16A.CEL

01p_HuGe1_marie_francoise_ritz_210912_.CEL

06p_HuGe1_marie_francoise_ritz_210912_.CEL

08p_HuGe1_marie_francoise_ritz_210912_.CEL

09p_HuGe1_marie_francoise_ritz_210912_.CEL

10p_HuGe1_marie_francoise_ritz_210912_.CEL

5_07_03_HuGe1_mf_ritz_140711_.CEL

5_08_04_HuGe1_mf_ritz_140711_.CEL

6_02_06_HuGe1_mf_ritz_140711_.CEL

6_03_07_HuGe1_mf_ritz_140711_.CEL

6_08_12_HuGe1_mf_ritz_140711_.CEL

7_01_13_HuGe1_mf_ritz_140711_.CEL

7_06_18_HuGe1_mf_ritz_140711_.CEL

7_07_19_HuGe1_mf_ritz_140711_.CEL

8_01_21_HuGe1_mf_ritz_140711_.CEL

8_02_22_HuGe1_mf_ritz_140711_.CEL

GSM1671354_Kidney_Benign_AX2489.CEL

GSM1671385_Kidney_Normal_AX1134.CEL

GSM1671386_Kidney_Normal_AX1254.CEL

GSM1671387_Kidney_Normal_AX1312.CEL

GSM1671388_Kidney_Normal_AX1549.CEL

GSM1671389_Kidney_Normal_AX1662.CEL

GSM1671390_Kidney_Normal_AX1664.CEL

GSM1671391_Kidney_Normal_AX1692.CEL

GSM1671392_Kidney_Normal_AX1824.CEL

GSM1671393_Kidney_Normal_AX2024.CEL

GSM1671394_Kidney_Normal_AX2150.CEL

GSM1671395_Kidney_Normal_AX2164.CEL

GSM1671396_Kidney_Normal_AX2218.CEL

GSM1671397_Kidney_Normal_AX0745.CEL

GSM1671398_Kidney_Normal_AX0992.CEL

GSM1708491_RSM06855.CEL

GSM1708492_RSM06856.CEL

GSM1708493_RSM06859.CEL

GSM1708494_RSM06860.CEL

GSM1708500_RSM09739.CEL

GSM1708501_RSM09740.CEL

GSM1708502_RSM09741.CEL

GSM1708503_RSM09742.CEL

GSM1708508_RSM10641.CEL

GSM1708509_RSM10642.CEL

GSM1708510_RSM10643.CEL

GSM1708511_RSM10644.CEL

GSM1708512_RSM10645.CEL

GSM1708513_RSM10646.CEL

GSM1426079_controle_colon_76.CEL

GSM1426080_controle_colon_77.CEL

GSM1426081_controle_colon_78.CEL

GSM1426082_controle_colon_79.CEL

GSM1426083_controle_colon_80.CEL

GSM1426084_controle_colon_81.CEL

GSM1426085_controle_colon_82.CEL

GSM1426086_controle_colon_83.CEL

GSM1426087_controle_colon_84.CEL

GSM1426088_controle_colon_85.CEL

GSM1426089_controle_colon_86.CEL

MV14.CEL

MV16.CEL

MV17.CEL

MV19.CEL

MV22.CEL

MV28.CEL

MV29.CEL

MV30.CEL

MV39.CEL

MV4.CEL

MV42.CEL

MV52.CEL

MV55.CEL

MV56.CEL

MV59.CEL

GSM2079479_01.UC137.CEL

GSM2079480_02.UC207.CEL

GSM2079481_03.UC251.CEL

GSM2079482_04.UC230.CEL

GSM2079483_05.UC182.CEL

GSM1326668_C1.CEL

GSM1326669_C2.CEL

GSM1326670_C3.CEL

GSM1422185_007N_HuGene-1_0-st-v1_.CEL

GSM1422187_014N_HuGene-1_0-st-v1_.CEL

GSM1422189_017N_HuGene-1_0-st-v1_.CEL

GSM1422191_018N_HuGene-1_0-st-v1_.CEL

GSM1422193_021N_HuGene-1_0-st-v1_.CEL

GSM1422195_033N_HuGene-1_0-st-v1_.CEL

GSM1422197_043N_HuGene-1_0-st-v1_.CEL

GSM1422199_054N_HuGene-1_0-st-v1_.CEL

GSM1422201_057N_HuGene-1_0-st-v1_.CEL

GSM1422203_066N_HuGene-1_0-st-v1_.CEL

GSM1422205_070N_HuGene-1_0-st-v1_.CEL

GSM1422209_075N_HuGene-1_0-st-v1_.CEL

GSM1422211_076N_HuGene-1_0-st-v1_.CEL

GSM1422213_077N_HuGene-1_0-st-v1_.CEL

GSM1245969_Milk_tooth1.CEL

GSM1245970_Milk_tooth2.CEL

GSM1245971_Milk_tooth3.CEL

GSM1245972_Adult_tooth1.CEL

GSM1245973_Adult_tooth2.CEL

GSM1245974_Adult_tooth3.CEL

GSM1435033_PR-AT1.CEL

GSM1435034_PR-AT2.CEL

GSM1435035_PR-AT3.CEL

GSM1435036_PR-AT4.CEL

GSM1435037_PR-AT5.CEL

GSM1435038_PR-AT6.CEL

GSM1435039_PR-AT7.CEL

GSM1435040_PR-AT8.CEL

GSM1435041_PR-AT9.CEL

GSM1435042_PR-AT10.CEL

GSM886560_TN-144-10R_HuGene-1_0-st-v1_.CEL

GSM886561_TN-661-11R_HuGene-1_0-st-v1_.CEL

GSM886562_TN-687-11R_HuGene-1_0-st-v1_.CEL

GSM886563_TN-690-11R_HuGene-1_0-st-v1_.CEL

GSM886564_TN-1412-09R-CONTROL_HuGene-1_0-st-v1_.CEL

GSM1311815_201247_HUGENE-1_0-ST-V1_11.CEL

GSM1311816_201249_HUGENE-1_0-ST-V1_13.CEL

GSM1311817_201250_HUGENE-1_0-ST-V1_14.CEL

GSM1311818_201251_HUGENE-1_0-ST-V1_15.CEL

GSM1311819_201253_HUGENE-1_0-ST-V1_17.CEL

GSM1311820_201254_HUGENE-1_0-ST-V1_18.CEL

GSM1311821_201255_HUGENE-1_0-ST-V1_19.CEL

GSM1311822_201257_HUGENE-1_0-ST-V1_21.CEL

GSM1311828_201263_HUGENE-1_0-ST-V1_27.CEL

GSM1311829_201264_HUGENE-1_0-ST-V1_28.CEL

GSM1311830_201265_HUGENE-1_0-ST-V1_29.CEL

GSM1311831_201266_HUGENE-1_0-ST-V1_30.CEL

GSM1311832_201267_HUGENE-1_0-ST-V1_31.CEL

GSM1311833_201268_HUGENE-1_0-ST-V1_32.CEL

01_H46.CEL

02_H50.CEL

03_H57.CEL

04_H59.CEL

05_H62.CEL

06_H69.CEL

07_H72_23_2.CEL

08_H73.CEL

09_H77.CEL

10_H84.CEL

11_H56.CEL

12_H64.CEL

13_H65.CEL

14_H66.CEL

15_H68.CEL

GSM1376743_A0044470.CEL

GSM1376750_A0044484.CEL

GSM1376751_A0044489.CEL

GSM1376753_A0044491.CEL

GSM1376754_A0044493.CEL

009A.CEL

014A.CEL

039A.CEL

GSM878376.CEL

GSM878384.CEL

GSM878385.CEL

GSM878389.CEL

GSM878391.CEL

GSM878395.CEL

GSM878397.CEL

GSM878401.CEL

GSM878409.CEL

GSM878410.CEL

GSM878417.CEL

GSM878419.CEL

GSM878420.CEL

GSM878423.CEL

GSM878425.CEL

GSM878221.CEL

GSM878226.CEL

GSM878230.CEL

GSM878236.CEL

GSM878239.CEL

GSM878240.CEL

GSM878242.CEL

GSM878245.CEL

GSM878252.CEL

GSM878257.CEL

GSM878258.CEL

GSM878259.CEL

GSM878260.CEL

GSM878261.CEL

GSM878263.CEL

GSM926974.CEL

GSM926975.CEL

GSM926976.CEL

GSM926977.CEL

GSM926978.CEL

GSM926979.CEL

GSM926980.CEL

GSM926981.CEL

GSM926982.CEL

GSM926983.CEL

GSM926984.CEL

GSM926985.CEL

GSM926986.CEL

GSM926987.CEL

GSM926988.CEL

GSM946253_2010-09-14_K1_HuGene-1_0-st-v1.CEL

GSM946254_2010-09-14_K3_HuGene-1_0-st-v1.CEL

GSM946255_2010-09-14_KL13_HuGene-1_0-st-v1.CEL

GSM946256_2010-09-14_KL14_HuGene-1_0-st-v1.CEL

GSM946257_2010-09-14_KL15_HuGene-1_0-st-v1_2.CEL

GSM946258_2010-09-14_KL16_HuGene-1_0-st-v1.CEL

GSM946259_2010-09-14_KL6_HuGene-1_0-st-v1.CEL

GSM953547_CEXO-10.CEL

GSM953548_CEXO-13.CEL

GSM953549_CEXO-19.CEL

GSM953550_CEXO-5.CEL

GSM953551_CEXO-7.CEL

GSM973621_2-N_518.CEL

GSM973622_2-N_511.CEL

GSM973623_2-N_504HIP.CEL

GSM973624_2-N_504.CEL

GSM973625_2-N_502.CEL

GSM973626_1-N_483.CEL

GSM973627_2-N_434.CEL

GSM973628_2-N_432.CEL

GSM1015738_G330_10.CEL

GSM1015739_G330_11.CEL

GSM1015740_G330_12.CEL

GSM1015741_G330_16.CEL

GSM1015742_G330_17.CEL

GSM1015743_G330_18.CEL

GSM1062805_GFS_IW00101.CEL

GSM1062806_GFS_IW00102.CEL

GSM1062807_GFS_IW00103.CEL

GSM1062808_GFS_IW00104.CEL

GSM1062809_GFS_IW00105.CEL

GSM1062810_GFS_IW00106.CEL

GSM1062811_GFS_IW00107.CEL

GSM1062812_GFS_IW00108.CEL

GSM1062813_GFS_IW00109.CEL

GSM1062814_GFS_IW00110.CEL

GSM1062815_GFS_IW00111.CEL

GSM1062816_GFS_IW00112.CEL

GSM1062817_GFS_IW00113.CEL

GSM1062818_GFS_IW00114.CEL

GSM1062819_GFS_IW00115.CEL

GSM1070451_EXSK1102_13.CEL

GSM1070453_EXSK1102_15.CEL

GSM1070455_EXSK1102_17.CEL

GSM1070457_EXSK1102_19.CEL

GSM1070459_EXSK1102_21.CEL

GSM1070461_EXSK1102_23.CEL

GSM1098778_Myomet0101_HuGene-1_0-st-v1_.CEL

GSM1098779_Myomet0102_HuGene-1_0-st-v1_.CEL

GSM1098780_Myomet0103_HuGene-1_0-st-v1_.CEL

GSM1108350_S1521_bone_marrow.CEL

GSM1108351_S1525_spleen.CEL

GSM1108352_S1701_thymus.CEL

GSM1108353_S1703_stomach.CEL

GSM1108354_S2086_MuAS-NG.CEL

GSM1108355_S1526_small_intestine.CEL

GSM1108356_S1702_colon_mucosa.CEL

GSM1108357_S1522_heart.CEL

GSM1108358_S1523_liver.CEL

GSM1108359_S1524_lung.CEL

GSM1108360_S1699_skeletal_muscle.CEL

GSM1108361_S1696_Brain.CEL

GSM1108362_S2156_Brain_cerebellum.CEL

GSM1108363_S2161_Spinal_cord.CEL

GSM1108364_S2159_Trachea.CEL

GSM1142130_UC8926.CEL

GSM1142131_UC9009.CEL

GSM1142132_UC9023.CEL

GSM1142133_UC9101.CEL

GSM1142134_UC9612.CEL

GSM1142135_UC9011.CEL

GSM1142136_UC9035.CEL

GSM1232077_EA1-HuGene-1_0-ST-9960.CEL

GSM1232078_EA1-HuGene-1_0-ST-9959.CEL

GSM1232079_EA1-HuGene-1_0-ST-9958.CEL

GSM1232080_EA1-HuGene-1_0-ST-9957.CEL

GSM1232081_EA1-HuGene-1_0-ST-9956.CEL

GSM1232082_EA1-HuGene-1_0-ST-9955.CEL

GSM1232083_EA1-HuGene-1_0-ST-9954.CEL

GSM1232084_EA1-HuGene-1_0-ST-9953.CEL

GSM1232085_EA1-HuGene-1_0-ST-9952.CEL

GSM1281638_Hs_A510_Normal.CEL

GSM1281639_Hs_A508_Normal.CEL

GSM1281640_Hs_A506_Normal.CEL

JLI_1_HuGene.CEL

JLI_11_HuGene.CEL

JLI_13_HuGene.CEL

JLI_23_HuGene.CEL

JLI_3_HuGene.CEL

JLI_39_HuGene.CEL

JLI_43_HuGene.CEL

JLI_45_HuGene.CEL

JLI_47_HuGene.CEL

JLI_49_HuGene.CEL

JLI_53_HuGene.CEL

JLI_59_HuGene.CEL

JLI_64_HuGene.CEL

JLI_66_HuGene.CEL

JLI_7_HuGene.CEL

GSM475621_201553_C1_Chami.CEL

GSM475622_627618_C2_Chami.CEL

GSM475623_627618_C3_Chami.CEL

GSM475624_201553_C4_Chami.CEL

GSM475631_627618_C5_Chami.CEL

GSM475633_627618_C6_Chami.CEL

GSM475638_201553_C11_Chami.CEL

GSM475642_201553_C7_Chami.CEL

GSM475645_627618_C8_Chami.CEL

GSM475648_201553_C9_Chami.CEL

GSM475649_627618_C10_Chami.CEL

GSM700796.CEL

GSM700797.CEL

GSM700798.CEL

GSM701161.CEL

GSM701162.CEL

GSM701163.CEL

GSM701164.CEL

GSM701165.CEL

GSM737013_CT_LSF1.CEL

GSM737014_CT_LSF2.CEL

GSM737015_CT_LSF3.CEL

GSM737016_CT_LSF4.CEL

GSM737017_CT_LSM1.CEL

GSM737018_CT_LSM2.CEL

GSM737019_CT_LSM3.CEL

GSM737020_CT_LSM4.CEL

GSM737021_CT_LVF1.CEL

GSM737022_CT_LVM1.CEL

GSM834032.CEL

GSM834033.CEL

GSM835234.CEL

GSM835235.CEL

GSM835236.CEL

GSM835237.CEL

GSM835238.CEL

GSM835239.CEL

GSM835241.CEL

GSM851993.CEL

GSM851994.CEL

GSM851995.CEL

GSM851996.CEL
