# Supplementary material for: Comprehensive Molecular Profiling for Relapsed/Refractory Pediatric Burkitt Lymphomas—Retrospective Analysis of Three Real-Life Clinical Cases—Addressing Issues on Randomization and Customization at the Bedside
Source: Front Oncol. 2020 Feb 7;9:1531. doi: 10.3389/fonc.2019.01531 (PMC7027364; doi:10.3389/fonc.2019.01531)
Supplement: Supplementary file 2 [file Data_Sheet_2.pdf]

Supplementary Material 2

Gene	Protein	Variant (c.DNA/protein)	F (%)	dbSNP ID	Transcript	Variant classification	Reference
ALK	ALK receptor tyrosine kinase	c.2867T>G/p.V956G	38	-	NM_004304	VUS (class 3)	novel
MSH2	mutS homolog 2	c.70_97del/p.Q24fs	31,8	-	NM_000251	VLP (class 4)	novel
MYC	MYC proto-oncogene	c.80A>T/p.Y27F	55,9	-	NM_002467	VUS (class 3)	novel
		c.380A>G/p.N127S	55,8	-		VUS (class 3)	novel
		c.566C>G/p.S189C	55,6	-		VUS (class 3)	novel
		c.791G>A/p.S264N	52,9	-		VUS (class 3)	Zhang et al, 2013
AXIN1	axin 1	c.1475G>A/p.R492H	41,4	rs765982081	NM_003502	VUS (class 3)	Kurihara et al, 2004
CREBBP	CREB binding protein	c.1823+2T>C	43,5	-	NM_004380	VLP (class 4)	novel

TP53	tumor protein p53	c.817C>T/p.R273C	85,8	rs121913343	NM_000546	VLP (class 4)	Ohgaki, Kleihues and Heitz, 1993
NF1	neurofibromin 1	c.7004C>T/p.P2335L	37,1	rs1555535402	NM_000267	VUS (class 3)	dbSNP
NOTCH1	notch 1	c.5678delG/p.G1893fs	26,7	-	NM_017617	VLP (class 4)	novel
NOTCH2	notch 2	c.3481G>A/p.G1161R	45,8	rs782313621	NM_024408	VUS (class 3)	Jones et al, 2014

Table Case 1: Selected variants found in somatic exome of Case 1. "F" indicates frequency of variant in the tumour sample. Classification of variants is according to ACMG guidelines. Tumour mutation burden (load) was 16 mutations/ Mb – intermediate/high[1–4].

**Gene**	**Protein**	**Variant (c.DNA/protein)**	**F (%)**	**dbSNP ID**	**Transcript**	**Variant classification**	**Reference**
CCND3	cyclin D3	c.869T>A/p.I290K	40	rs1354292944	NM_001760	VUS (class 3)	Pasqualucci et al, 2011

TP53	tumor protein p53	c.743G>T/p.R248L	80,3	rs11540652	NM_000546	VLP (class 4)	Chevillard et al, 1997
SMARCA4	SWI/SNF related, matrix associated, actin dependent regulator of chromatin, subfamily a, member 4	c.3745G>C/p.A1249P	39,5	-	NM_003072	VUS (class 3)	novel
FBXW7	F-box and WD repeat domain containing 7	c.1703T>G/p.L568R	31,8	-	NM_018315	VUS (class 3)	novel

Table Case 2: Selected variants found in somatic exome of Case 2. "F" indicates frequency of variant in the tumour sample. Classification of variants is according to ACMG guidelines. Tumour mutation burden (load) was 8 mutations/ Mb – intermediate[5,6].

Gene	Protein	Variant (c.DNA/protein)	F (%)	dbSNP	Transcript	Variant classification	Reference
MYC	MYC proto-oncogene	c.17T>C/p.V6A	29	-	NM_002467	VUS (class 3)	novel
CDKN2A	cyclin dependent kinase inhibitor 2A	c.238C>T/p.R80*	64	rs121913388	NM_000077	VLP (class 4)	Zhou X et al, 1994
CDC73	cell division cycle 73	c.530T>C/p.M177T	39	-	NM_024529	VUS (class 3)	novel
NF1	neurofibromin 1	c.1765C>T/p.Q589*<br>c.2156T>C/p.I719T<br>c.2507A>C/p.E836A	5<br>10<br>10	rs1282299543<br>rs754025938<br>-	NM_000267	VLP (class 4)<br>VUS (class 3)<br>VUS (class 3)	Nik-Zainal S et al, 2016<br>dbSNP<br>novel
NOTCH1	notch receptor 1	c.2806G>T/p.G936C	8	-	NM_017617	VUS (class 3)	TCGA

PTPN11	protein tyrosine phosphatase non-receptor type 11	c.185A>G/p.Y62C	8	rs1013419211	NM_002834	VUS (class 3)	dbSNP

Table Case 3: Selected variants found in somatic exome of Case 3. "F" indicates frequency of variant in the tumour sample. Classification of variants is according to ACMG guidelines. Tumour mutation burden (load) was 31 mutations/ Mb – high[7,8].

References Supplementary Material 2:

1. Jones S, Stransky N, McCord CL, et al. Genomic analyses of gynaecologic carcinosarcomas reveal frequent mutations in chromatin remodelling genes. Nat Commun 2014;5(1):5006.

2. Kurihara T, Ikeda S, Ishizaki Y, et al. Immunohistochemical and Sequencing Analyses of the Wnt Signaling Components in Japanese Anaplastic Thyroid Cancers. Thyroid 2004;14(12):1020–1029.

3. Zhang J, Grubor V, Love CL, et al. Genetic heterogeneity of diffuse large B-cell lymphoma. Proc Natl Acad Sci USA 2013;110(4):1398–1403.

4. Ohgaki H, Kleihues P, Heitz PU. p53 Mutations in sporadic adrenocortical tumors. Int J Cancer 1993;54(3):408–410.

5. Pasqualucci L, Trifonov V, Fabbri G, et al. Analysis of the coding genome of diffuse large B-cell lymphoma. Nat Genet 2011;43(9):830–837.

6. Chevillard S, Lebeau J, Pouillart P, et al. Biological and clinical significance of concurrent p53 gene alterations, MDR1 gene expression, and S-phase fraction analyses in breast cancer patients treated with primary chemotherapy or radiotherapy. Clin Cancer Res 1997;3(12 Pt 1):2471–2478.

7. Zhou X, Tarmin L, Yin J, et al. The MTS1 gene is frequently mutated in primary human esophageal tumors. Oncogene 1994;9(12):3737–3741.

8. Nik-Zainal S, Davies H, Staaf J, et al. Landscape of somatic mutations in 560 breast cancer whole-genome sequences. Nature 2016;534(7605):47–54.
